# Pathogenic Variants in *ACTRT1* Cause Acephalic Spermatozoa Syndrome

**DOI:** 10.3389/fcell.2021.676246

**Published:** 2021-08-06

**Authors:** Yanwei Sha, Wensheng Liu, Lin Li, Mario Serafimovski, Vladimir Isachenko, Youzhu Li, Jing Chen, Bangrong Zhao, Yifeng Wang, Xiaoli Wei

**Affiliations:** ^1^Department of Andrology, United Diagnostic and Research Center for Clinical Genetics, School of Public Health & Women and Children’s Hospital, Xiamen University, Xiamen, China; ^2^Obstetrics and Gynecology Center, Department of Obstetrics and Gynecology, Zhujiang Hospital, Southern Medical University, Guangzhou, China; ^3^Research Group for Reproductive Medicine, Department of Obstetrics and Gynecology, Medical Faculty, University of Cologne, Cologne, Germany; ^4^Central Laboratory, Beijing Obstetrics and Gynecology Hospital, Capital Medical University, Beijing, China; ^5^Center for Physiology and Pathophysiology, Medical Faculty, University of Cologne, Cologne, Germany; ^6^Reproductive Medicine Center, The First Affiliated Hospital of Xiamen University, Xiamen, China; ^7^NHC Key Laboratory of Family Planning and Healthy/Key Laboratory of Reproductive Medicine of Hebei Provincial, Shijiazhuang, China; ^8^State Key Laboratory of Cellular Stress Biology, School of Pharmaceutical Sciences, Xiamen University, Xiamen, China

**Keywords:** acephalic spermatozoa syndrome, whole-exome sequencing, *ACTRT1*, pathogenic variants, teratozoospermia

## Abstract

Acephalic spermatozoa syndrome is a rare type of teratozoospermia, but its pathogenesis is largely unknown. Here, we performed whole-exome sequencing for 34 patients with acephalic spermatozoa syndrome and identified pathogenic variants in the X-linked gene, *ACTRT1*, in two patients. Sanger sequencing confirmed the pathogenic variants of *ACTRT1* in the patients. Both pathogenic variants of *ACTRT1* were highly conserved, and *in silico* analysis revealed that they were deleterious and rare. *Actrt1*-knockout mice exhibited a similar acephalic spermatozoa phenotype. Therefore, we speculated that mutations in *ACTRT1* account for acephalic spermatozoa syndrome. Moreover, the patients in this study conceived their children through artificial insemination. This study provides further insights for clinicians and researchers regarding the genetic etiology and therapeutic strategies for acephalic spermatozoa patients with pathogenic variants in *ACTRT1*.

## Introduction

Acephalic spermatozoa syndrome (MIM: 617187) is a rare type of teratozoospermia that severely affects male fertility (Chemes et al., 1987). There is no large-scale epidemiological survey on the incidence of acephalic spermatozoa syndrome, but it is speculated that the incidence of acephalic spermatozoa syndrome may be less than 0.1% based on the reported cases (Li et al., 2019). Because of the low proportion of patients with acephalic spermatozoa syndrome in the population, this particular syndrome has drawn little attention and related reports are very limited. Moreover, most of the patients with acephalic spermatozoa syndrome encountered clinically do not have completely headless sperm; thus, clinicians can easily ignore patients with partial acephalic spermatozoa syndrome. Therefore, there is only limited research on the pathogenesis of acephalic spermatozoa syndrome (Jiao et al., 2021). Considering the severe effects of acephalic spermatozoa syndrome on male reproductive and mental health, there is an urgent need to comprehensively and systematically study the pathogenesis of acephalic spermatozoa syndrome, to further guide the clinical screening and treatment of this disorder.

The occurrence of acephalic spermatozoa syndrome is characteristic of idiopathic, familial aggregation; therefore, genetic defects may be the main cause of this disease (Lu et al., 2021). Several studies have shown that genetic defects are an important cause of acephalic spermatozoa syndrome (Baccetti et al., 1989; Wu et al., 2020). However, previous studies have only found a few genes associated with human acephalic spermatozoa syndrome, such as *SUN5*, *PMFBP1*, *TSGA10*, *BRDT*, *HOOK1*, *DNAH6*, and *CEP112* (Li et al., 2017, 2018; Chen et al., 2018; Sha et al., 2018a,b, 2019, 2020; Ye et al., 2020; Mazaheri Moghaddam et al., 2021). As the head-tail coupling apparatus (HTCA) of spermatozoa is complex and consists of multiple proteins, current knowledge of the causes of acephalic spermatozoa syndrome may only be the tip of the iceberg (Wu et al., 2020). Defects in *SUN5* or *PMFBP1* are the main causes of acephalic spermatozoa syndrome, accounting for approximately 30% of cases. However, the pathogenesis of the syndrome in the remaining patients remains largely unknown, and more research is imperative to fully elucidate the pathogenesis of acephalic spermatozoa syndrome.

Actin-related protein T1 (ACTRT1, also known as ARPT1), which is encoded by *ACTRT1* (Gene ID: 139741), is specifically expressed in the testes. The insertion mutation, c.547_548insA, in *ACTRT1* has been identified in two of six families with Bazex-Dupré-Christol syndrome, suggesting that mutations in *ACTRT1* are likely to cause human disease (Bal et al., 2017). Heid et al. (2002) used immunofluorescence staining to show that ACTRT1 is mainly expressed in the calyx and pericentriolar material (PCM) of bull spermatozoa (Heid et al., 2002). Centrioles are an indispensable component of the HTCA, and the dysfunction of sperm centriole-associated proteins can cause acephalic spermatozoa syndrome (Chemes and Rawe, 2010; Kim et al., 2018; Reina et al., 2018; Sha et al., 2020). These data indicate that *ACTRT1* may play a role in fastening the sperm head and flagellum.

In this study, we used whole-exome sequencing (WES) to identify pathogenic variants in two patients with acephalic spermatozoa syndrome. Both variants were deleterious and may affect the function and stability of ACTRT1. *Actrt1*-knockout mice exhibited a similar acephalic spermatozoa phenotype. Therefore, our results indicated that *ACTRT1* is essential for the normal formation of the sperm head-tail junction, and defects in this gene may be involved in the etiology of acephalic spermatozoa syndrome.

## Materials and Methods

### Patients and Control Subjects

We performed WES of 34 patients with acephalic spermatozoa syndrome. Patients with partial acephalic spermatozoa syndrome (proportion of headless sperm greater than 50%) were included in the study. Patients who had a childhood disease, environmental or radiation exposure, prescription drug usage, abnormal somatic karyotypes, microdeletions on the Y chromosome, or other non-genetic factors that may contribute to male infertility were excluded from the study. Thirty healthy men with proven reproductive capacity served as control subjects. All participants underwent a physical examination. Routine semen analysis was performed according to the guidelines of the World Health Organization Laboratory Manual for the Examination and Processing of Human Semen (fifth edition). We obtained 5 mL of peripheral blood for reproductive hormone and WES analysis, semen for further research, and written informed consent from each individual. This study was conducted in accordance with the 1964 Helsinki Declaration and its later amendments or comparable ethical standards. The study was approved by the Ethics Committees of the Women and Children’s Hospital of Xiamen University, the First Affiliated Hospital of Xiamen University, and Beijing Obstetrics and Gynecology Hospital.

### Whole-Exome Sequencing and Sanger Sequencing

Whole-exome sequencing and data analysis were performed by Annoroad Gene Technology Co., Ltd. (Beijing, China) according to the manufacturer’s instructions. Briefly, genomic DNA was extracted from peripheral blood samples and sequenced on a HiSeq 2500 platform (Illumina, San Diego, CA, United States). The reads were aligned to the human reference sequence (hg19) using Burrows-Wheeler Aligner software (Li and Durbin, 2009) and sorted using Picard tools^1^. Based on the results of the alignment, single nucleotide variants and indels were analyzed and quality-filtered using Genome Analysis Toolkit^2^ (McKenna et al., 2010). Candidate variants were annotated using ANNOVAR^3^ (Wang et al., 2010). Filter-based annotation was performed using SIFT^4^ (Ng and Henikoff, 2003; Vaser et al., 2016); PolyPhen-2^5^ (Adzhubei et al., 2013); MutationTaster^6^ (Schwarz et al., 2010); gnomAD^7^ (Koch, 2020); and other databases. Variants satisfying the following criteria were retained for subsequent analyses: (1) absent or rare variants; (2) nonsense, frameshift, splice-site, or missense variants. Testicular-specific genes that met the above screening criteria, especially those expressed in the head-tail junction of sperm, were considered as a priority. Sanger sequencing was used to verify mutations in patients and their family members. The primers used for Sanger sequencing are listed in Supplementary Table 1.

### Papanicolaou Staining

Papanicolaou staining of the spermatozoa was performed according to the World Health Organization Laboratory Manual for the Examination and Processing of Human Semen (fifth edition) with modifications to confirm morphological changes in sperm flagella (Liu et al., 2019). Briefly, slides were fixed in 95% ethanol and then immersed in a gradient of alcohol solutions from 80 to 50%. The slides were then rinsed with distilled water, stained with hematoxylin. After soaking with distilled water and ethanol hydrochloride. After dehydration dehydrated in a gradient of alcohol solutions from 50 to 90%, the samples were stained with Orange G6 and EA50 and then dehydrated in 95% alcohol. Subsequently, the slides were washed in xylene and mounted with a permanent mounting medium.

### Protein Structural Analysis

Structural analysis of mutant ACTRT1 proteins (NP_612146.1) was performed using SWISS-MODEL software^8^ based on the template protein structure of Actin-1 from SMTL (ID: 4efh.1). The protein structures were visualized and the structure was visualized using SWISS-PdbViewer 4.1.0^9^ software.

### Immunostaining of Spermatozoa

Immunostaining of spermatozoa was performed as previously described (Sha et al., 2017). Briefly, the prepared spermatozoa were smeared onto poly-L-lysine-coated slides, air-dried, washed in phosphate-buffered saline (PBS), fixed in 4% paraformaldehyde (F8775; Sigma, St Louis, MO, United States) for 10 min at room temperature, washed twice in PBS, and permeabilized with 0.2% Triton X-100 (93443, Sigma). The samples were blocked for 30 min at room temperature. Slides were incubated with primary antibodies for 1 h at room temperature, followed by incubation with Alexa Fluor^®^ 594-conjugated goat anti-rabbit IgG (ZF-0516; Zsbio, Beijing, China) and Alexa Fluor^®^ 488-conjugated goat anti-mouse IgG (ZF-0512, Zsbio) secondary antibodies for 1 h at RT. Slides were subsequently washed three times in PBS, mounted with Vectorshield containing 4′,6-diamidino-2-phenylindole (H-1200; Vector Laboratories, Burlingame, CA, United States) and examined under a laser-scanning confocal immunofluorescence microscope (LSM780; Carl Zeiss, Oberkochen, Germany). The primary antibodies used in this study were anti-ACTRT1 (A57994; EpiGentek, New York, NY, United States), anti-PCM1 (sc-50164; Santa Cruz, Dallas, TX, United States), and anti-acetylated tubulin (66200-1-Ig, Proteintech Group, Chicago, IL, United States).

### Immunoblotting Analysis

Samples were lysed using RIPA lysis buffer, and protein concentrations were determined using a BCA Protein Assay Kit (23227; Thermo Fisher, Waltham, MA, United States). The proteins were extracted and separated by 10% (w/v) sodium dodecyl sulfate-polyacrylamide gel electrophoresis and transferred to polyvinylidene difluoride membranes (IPVH00010; Millipore, Billerica, MA, United States). Membranes were blocked for 1 h at room temperature with 5% skimmed milk in Tris-buffered saline solution (pH 7.4) containing 0.05% Tween-20 (TBST), and then incubated with anti-ACTRT1 (orb252388; Biorbyt, Cambridge, United Kingdom), anti-TSGA10 (12593-1-AP; Proteintech Group, Rosemont, IL, United States), anti-BRDT (AP7115a; Abgent, San Diego, CA, United States), or anti-acetylated tubulin (66200-1-Ig, Proteintech) primary antibodies overnight at 4°C. After washing three times with TBST, the membranes were incubated with horseradish peroxidase-conjugated goat anti-rabbit IgG secondary antibody (31460, Thermo Fisher) or goat anti-mouse IgG secondary antibody (31430, Thermo Fisher) for 1 h and washed three times with TBST at room temperature. The signals were developed using an enhanced chemiluminescence kit (K-12045-D50; Advansta, San Jose, CA, United States) and visualized and recorded using an ImageQuant LAS 4000 instrument (GE Healthcare Life Sciences, Marlborough, MA, United States).

### *In vitro* Fertilization Treatment

Assisted reproductive technology was performed in patients with *ACTRT1* mutations. For sperm optimization, the liquid semen of the patients was processed using the gradient centrifugation method in SpermGrad (10102; Vitrolife, Goteborg, Sweden) using a ROTOFIX 32A Centrifuge (Z601438; Hettich, Westphalia, Germany) at 300 × *g* for 15 min at room temperature. Treated semen samples with optimized sperm were then injected into the uterine cavity of the patients’ wives through a fertilizing tube. Clinical pregnancy was defined as the presence of a visible sac with a fetal heartbeat.

### Generation of *Actrt1*-Knockout Mouse Model

The *Actrt1*-knockout mouse model was generated using the transgenic platform of the Laboratory Animal Centre of Xiamen University. CRISPR/Cas9-mediated genome editing was performed according to our previously published protocol (Sha et al., 2019). We designed double gRNA primers to knock out a large fragment of the *Actrt1* exon. The gRNAs to knockout *Actrt1* (gRNA1, 5′-GACTAGGAACAACTGAGGTGC-3′; gRNA2, 5′-TGTGTCCCTCAGCATCCAAA-3′) were synthesized by Sangon Biotech Co., Ltd. (Shanghai, China). gRNA1 and gRNA2 were microinjected together with *Cas9* mRNA into the zygotes of C57BL/6 mice. Genotypes of the resulting pups were determined by Sanger sequencing. The founder mice, homozygous or heterozygous for the *Actrt1* knockout, were backcrossed onto a C57BL/6 background for at least two generations, and the resulting *Actrt1*^–/–^ mice were used in our experiments. All experiments involving mice were performed according to the methods approved by the Animal Ethics Committee of Xiamen University.

### Assay of Reproductive Ability

Fertility tests were performed by mating one 10-week-old *Actrt1*^+/+^ or *Actrt1*^–/–^ male with one 10-week-old wild-type female mouse (C57BL/6). Female mice with vaginal plugs were then moved into a separate cage and observed until the pups were born. 1 week later, male mice were mated with wild-type female mice. This mating experiment was repeated five times for each male mouse. All females were monitored during pregnancy. The dates of birth and the number of pups were recorded for all litters.

### Analyses of Mouse Headless Sperm Count and Morphology

Ten-week-old mice were sacrificed by cervical dislocation. Epididymides, along with the vas deferens, were dissected and cut into small pieces in a tube containing 1 mL Dulbecco’s modified Eagle medium, and then released into the medium during incubation at 37°C and 5% CO_2_ in a humidified incubator for 30 min. Headless sperm counts were determined using a hemocytometer for six mice per group, two hundred sperm were counted per mouse. Sperm morphology was observed under a microscope (BX53; Olympus, Tokyo, Japan) after unfixed sperm were spread onto precoated slides.

### Transmission Electron Microscopy

Transmission electron microscopy (TEM) was performed at the core facility of biomedical sciences of Xiamen University as described elsewhere (Liu et al., 2019). Briefly, the fresh spermatozoa were fixed in 2.5% glutaraldehyde before being washed twice in 0.1M phosphate buffer and resuspended in 0.2M sodium cacodylate buffer. These samples were then embedded in Epon 812 and then the ultrathin sections were stained with uranyl acetate and lead citrate before visualization via TEM (JEM-1400, Jeol, Japan).

### Label-Free Quantitative Proteomic Analyses

Label-free quantitative proteomic analyses were performed by Shanghai Applied Protein Technology Co. Ltd. (Shanghai, China) as described previously (Sha et al., 2019). In detail, germ cells were lysed in SDT buffer (containing 4% [w/v] SDS, 100 mM Tris/HCl [pH 7.6], and 0.1M DTT), and protein was quantified using a BCA Protein Assay Kit (23227, Thermo Fisher Scientific). Each sample was processed using the filter-aided proteome preparation method. Peptides were desalted using a C18 cartridge and resuspended in 0.1% formic acid. Samples were analyzed by LC-MS/MS using an Easy nLC system (Thermo Fisher). Trapping was performed using a nanoViper C18 column (Thermo Fisher, Acclaim PepMap100, 100 μm × 2 cm). Elution was performed on a Thermo Scientific C18-A2 EASY column (10 cm long, 75 μm i.d., 3 μm). Analytical separation of peptides was achieved at a flow rate of 300 nL/min using a Q-Exactive mass spectrometer. The dynamic exclusion time window was set at 60 s. Data were acquired with a normalized collision energy of 30 eV. Quantitative analysis was performed using MaxQuant software (version 1.5.3.17, Max Planck Institute of Biochemistry, Munich, Germany).

## Results

### Identification of *ACTRT1* Pathogenic Variants in Two Patients With Acephalic Spermatozoa Syndrome

**FIGURE 1:**
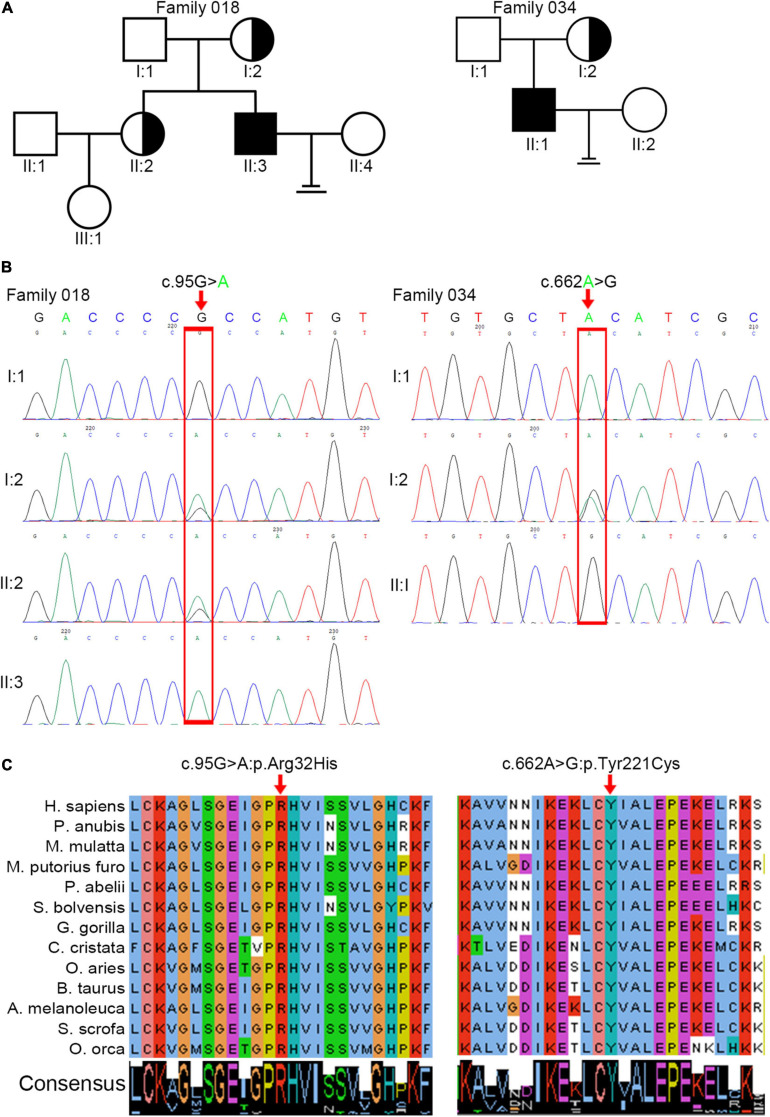
Identification of pathogenic variants in *ACTRT1* from two patients with acephalic spermatozoa syndrome. **(A)** Pedigree chart of the two patients harboring *ACTRT1* variants. The black squares represent the patients. **(B)** Sanger sequencing verified the pathogenic variants in the two patients. The mutation sites are indicated by the red rectangles. **(C)** Alignment analysis of the ACTRT1 amino acid sequences in different species in the affected sites, p.Arg32 and p.Tyr221.

We analyzed WES data from 34 patients with acephalic spermatozoa syndrome and found that *ACTRT1* was the most likely candidate gene for two of the patients (F018/II:3 and F034/II:1). Both patients were Han Chinese and came from unrelated non-consanguineous families (Figure 1A). The semen parameters of these two patients are presented in Table 1.

**TABLE 1:** Semen parameters of the patients with pathogenic variants in AC*TRT1*.

Patient	Semen volume (ml)	Semen pH	Sperm concentration (10^6^/ml)	Percentage of progressive motility (%)	Percentage of normal sperm (%)	Percentage of acephalic spermatozoa (%)
F018/II:3	2.77 ± 0.35	7.4	25.23 ± 7.62	18.20 ± 6.07	1.33 ± 0.58	68.33 ± 7.64
F034/II:1	3.23 ± 0.25	7.4	28.53 ± 8.30	22.33 ± 5.58	1.67 ± 0.58	62.67 ± 6.81
Reference	≥ 1.5	7.2∼7.8	≥ 15	≥ 32	≥ 4	≤ 10

We performed Sanger sequencing to verify the *ACTRT1* mutations in these two patients and their family members. The pathogenic variant, c.95G>A:p.Arg32His was detected in F018/II:3 and his mother (F018/I:2), and his older sister (F018/II:2), who had a daughter, was heterozygous for this variant. However, his father (F018/I:1) harbored the wild-type allele. The pathogenic variant, c.662A>G:pTyr221Cys was also detected in F034/II:1. His mother was heterozygous for this variant and his father harbored the wild-type allele (Figure 1B). Both pathogenic variants of *ACTRT1* had phenotypic consequences and showed familial segregation in a Mendelian manner.

### *In silico* Analysis of Pathogenic Variants in *ACTRT1*

The *ACTRT1* transcript (NM_138289.4) has only one exon and encodes the 376-amino acid protein, ACTRT1 (NP_612146.1). Both mutated sites appeared in the exon and cause amino acid substitutions. Predictions from SIFT, Polyphen-2_HDIV, Polyphen-2_HVAR, and PROVEAN databases suggested that both variants were highly pathogenic (Table 2). Neither variant was recorded in the total or East Asian populations in the gnomAD_genome database (Table 2). In addition, we evaluated the evolutionary conservation of ACTRT1. We aligned the amino acid sequences of ACTRT1 from different species and found that the amino acids affected by the pathogenic variants were highly conserved amongst different species (Figure 1C).

**TABLE 2:** *In silico* analysis of the pathogenic variants in *ACTRT1*.

Patient	Mutation	SIFT	Polyphen-2_HDIV	Polyphen-2_HVAR	PROVEAN	gnomAD_genome (All)	gnomAD_genome (EA)
F018/II:3	c.95G>A:p.Arg32His	Damaging (0.007)	Probably damaging (1)	Probably_damaging (0.984)	Damaging (−2.92)	0	0
F034/II:1	c.662A>G:p.Tyr221Cys	Damaging (0)	Possibly damaging (0.999)	Probably_damaging (0.973)	Damaging (−7.46)	NA	NA

**FIGURE 2:**
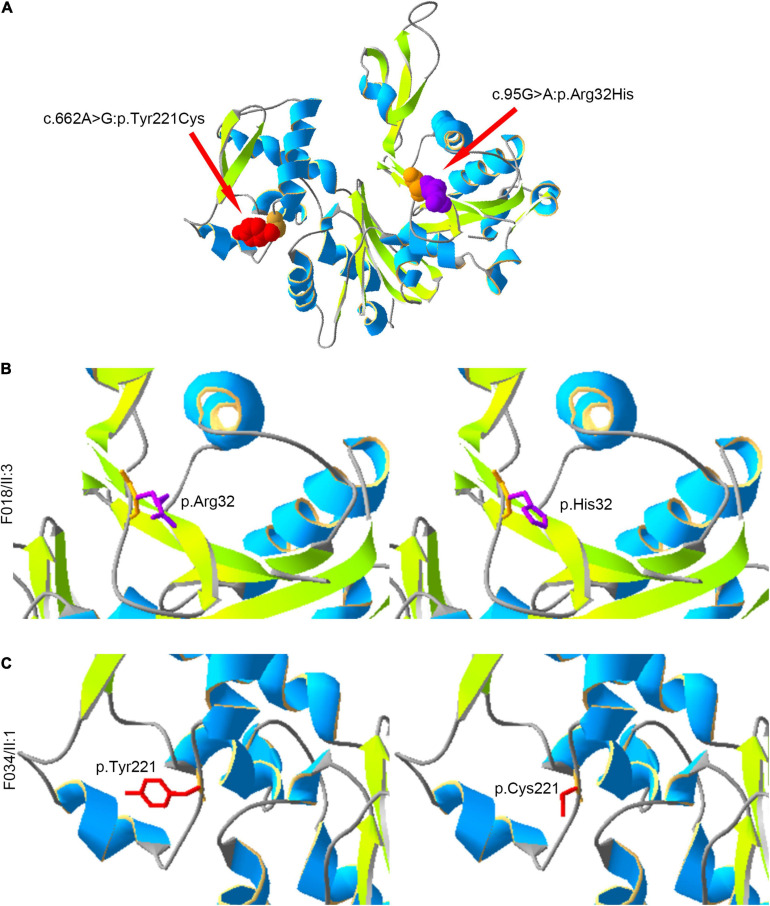
Locations of the amino acids affected by pathogenic variants in the three-dimensional structure of ACTRT1. **(A)** The positions of the amino acid substitutions in the three-dimensional structure of the original ACTR1 protein. **(B)** The pathogenic variant c.95G>A:p.Arg32His effect on the three-dimensional structure of ACTRT1. **(C)** The pathogenic variant c.662A>G:p.Tyr221Cys effect on the three-dimensional structure of ACTRT1.

Moreover, we constructed the mutated ACTRT1 protein structure using SWISS-MODEL. The amino acid substitutions caused by pathogenic variants of *ACTRT1* are shown in the three-dimensional protein structure in Figure 2A. The c.95G>A pathogenic variant identified in patient F018/II:3 caused the substitution of Arg to His at the 32nd amino acid of ACTRT1 (Figure 2B). Analogously, the c.662A>G pathogenic variant identified in patient F034/II:1 caused a substitution from Tyr to Cys at the 221st amino acid of ACTRT1 (Figure 2C). These variants may affect the three-dimensional structure of ACTRT1, which in turn may affect its stability and function.

### Patients With *ACTRT1* Mutations Exhibited Acephalic Spermatozoa Syndrome

We performed further detailed physical examinations of the patients with pathogenic *ACTRT1* variants and found that both patients had normal development of external genitalia and bilateral testicles. We did not detect any defects in the patients’ bilateral spermatic veins. Both patients had normal chromosomal karyotypes and hormone levels. The clinical data of the two patients with *ACTRT1* mutations patients are summarized in Supplementary Table 2.

**FIGURE 3:**
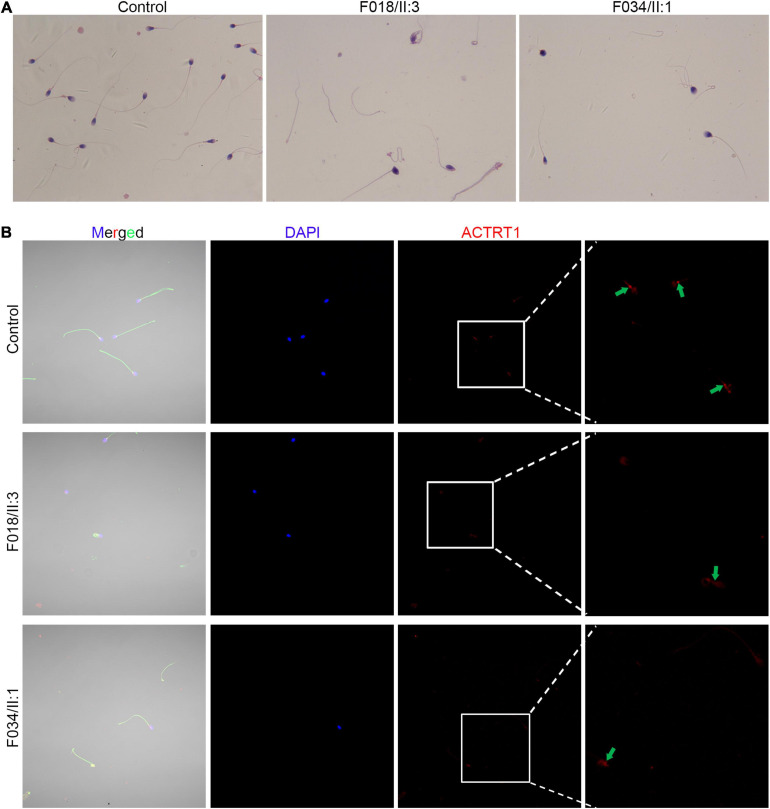
Pathogenic variants of *ACTRT1* caused acephalic spermatozoa syndrome. **(A)** Morphological analysis of spermatozoa from patients with *ACTRT1* mutations. Control subjects’ spermatozoa exhibited normal morphology, with the head and tail closely linked, while more than half of the patients’ spermatozoa were headless. **(B)** Immunofluorescence staining of ACTRT1 on the spermatozoa from control subjects and the patients.

Sperm morphology in patients with *ACTRT1* mutations was analyzed using Papanicolaou staining. The control subjects’ spermatozoa exhibited normal morphology with the head and the tail closely linked. However, most of the patients’ spermatozoa were headless, and a single head without a tail was observed at a low frequency (Figure 3A), resulting in a diagnosis of acephalic spermatozoa. We further analyzed the expression and localization of ACTRT1 by co-staining with PCM1 (a well-known pericentriolar protein) and found that it was highly expressed in the PCM of the sperm from control subjects (Supplementary Figure 1). However, in spermatozoa from affected patients, positive staining for ACTRT1 was dislocated and diffused (Figure 3B).

### *ACTRT1*-Knockout Male Mice Showed an Acephalic Spermatozoal Phenotype

**FIGURE 4:**
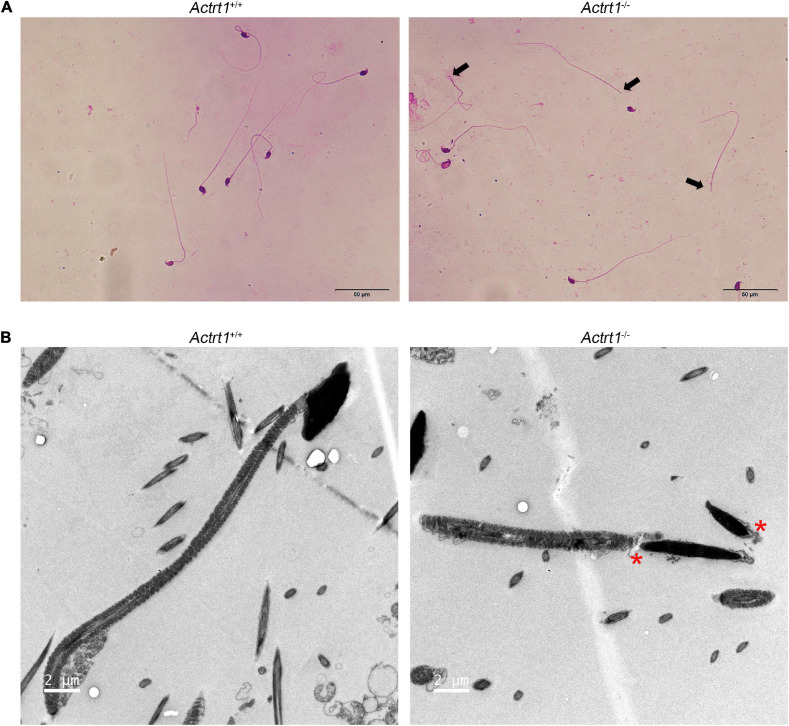
*Actrt1*-knockout mice showed a parallel acephalic spermatozoa phenotype. **(A)** Morphological analysis of spermatozoa from *Actrt1*-knockout mice. Spermatozoa from *Actrt1*-knockout mice were partially headless. Scale bar: 50 μm. **(B)** Ultrastructure of the sperm from *Actrt1*^+/+^ and *Actrt1*^–/–^ mice. Scale bar: 2 μm.

To further confirm the roles of *ACTRT1* in spermatogenesis and head-tail connection, we generated an *Actrt1*-knockout mouse model using CRISPR/Cas9-mediated genome editing (Supplementary Figure 2A). The results of PCR (Supplementary Figure 2B) and western blotting (Supplementary Figure 2C) confirmed that Actrt1 was successfully knocked out in mice. Hematoxylin and eosin staining showed normal spermatogenesis in the seminiferous tubules of *Actrt1*-knockout mice (Supplementary Figure 3A). However, a large part of the spermatozoa from *Actrt1*-knockout mice was headless (Supplementary Figure 3B and Figure 4A), further statistical data showed that about 60% spermatozoa from *Actrt1*-knockout mice was headless [*t*(5.949) = 10.422, *p* < 0.0001; Supplementary Figure 4]. As indicated by the red “*”, ultrastructural analysis by TEM showed that the HTCA of sperm from the *Actrt1*^–/–^ mice had significant defects (Figure 4B). Moreover, the fertility of male mice was severely impaired (Supplementary Table 3).

**FIGURE 5:**
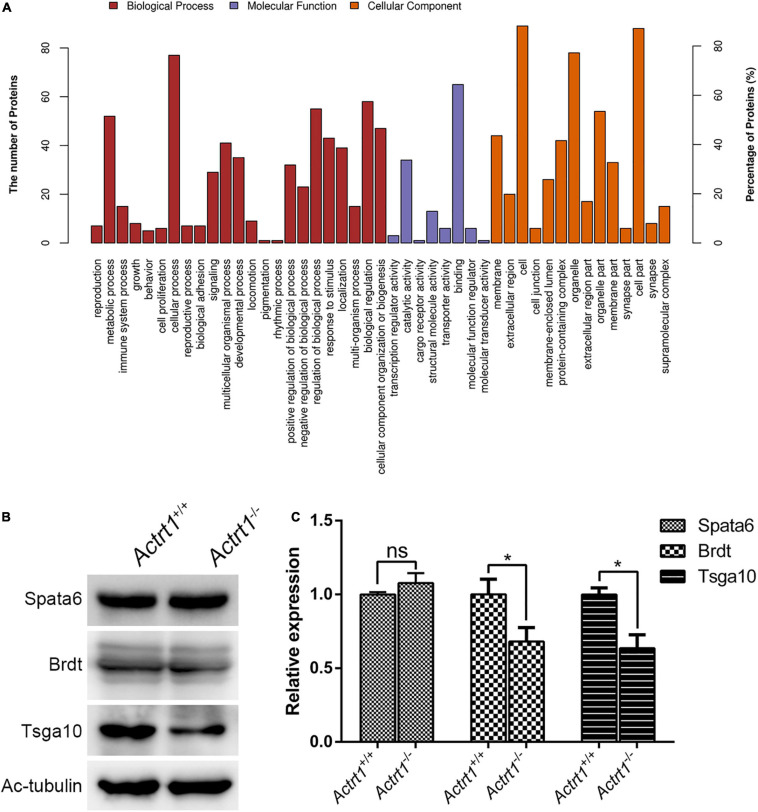
Label-free quantitative proteomics analysis of the testes from Actrt1-null mice. **(A)** Gene ontology analysis of the changed proteins from the testis of Actrt1-knockout mice. **(B)** Western blot analysis the expression of the proteins associated with acephalic spermatozoa syndrome. **(C)** The quantitative results from three independent experiments as described in **(B)**. *P < 0.05.

We performed label-free quantitative proteomics of the testis from *Actrt1-*knockout mice. The results showed that the levels of approximately 34 proteins were increased and the levels of 63 proteins were decreased by more than two times (Supplementary Table 4). Gene ontology analysis showed that these proteins were enriched in reproduction, cellular component organization or biogenesis, cell binding, and regulation of transport (Figure 5A). We performed Western blotting using sperm from *Actrt1*-knockout mice and found that the expression level of Spata6 was not affected. However, the expression levels of the potential genes associated with acephalic spermatozoa, *Tsga10* and *Brdt*, were reduced significantly (Figures 5B,C), which is consistent with the changes in acephalic spermatozoa-related gene expression levels seen in the label-free quantitative proteomics data (Supplementary Table 5).

### Clinical Pregnancy by Artificial Insemination

Given that the patients’ spermatozoa were partially headless, we optimized their spermatozoa and then performed artificial insemination for patients F018/II:3 and F034/II:1. Both procedures resulted in successful pregnancies and childbirth.

## Discussion

The most drastic morphological change during the process of sperm production is sperm spermiogenesis, which is a complex and highly ordered biological process (Hermo et al., 2010), in which HTCA is formed. The HTCA tightly concatenates the head and the tail of the sperm to maintain its integrity (Fawcett and Phillips, 1969). The formation of the HTCA is an intricate and complex process; thus, any defect in the assembly of HTCA or its surrounding structures may result in an abnormal HTCA, affect the connection of the sperm head and tail, and lead to acephalic spermatozoa syndrome (Wu et al., 2020).

Acephalic spermatozoa syndrome is a rare but severe form of teratozoospermia that seriously affects male reproduction (Perotti et al., 1981). Genetic defects have been shown to be the main pathogenic mechanism of acephalic spermatozoa syndrome (Baccetti et al., 1989; Wu et al., 2020; Lu et al., 2021). Defects in *SUN5* and *PMFBP1* are the most common etiologies of human acephalic spermatozoa syndrome, as identified by different investigators and demonstrated in a mouse model (Zhu et al., 2016, 2018; Elkhatib et al., 2017; Shang et al., 2017; Sha et al., 2018b, 2019; Liu et al., 2020; Lu et al., 2021; Zhang et al., 2021). *TSGA10* defects have also been reported to cause acephalic spermatozoa syndrome (Sha et al., 2018a; Liu et al., 2020; Ye et al., 2020). *BRDT*, *DNAH6*, *CEP112*, and *HOOK1* have also been reported to be associated with acephalic spermatozoa syndrome (Li et al., 2017, 2018; Chen et al., 2018; Sha et al., 2020). Compared to the few acephalic spermatozoa syndrome-related genes found in humans, genes associated with acephalic spermatozoa syndrome are more abundant in animal models. This may be because patients in the clinic most commonly present with partial acephalic spermatozoa syndrome. However, previous studies on acephalic spermatozoa syndrome have mainly focused on patients with more than 95% of decapitated spermatozoa, and there is a lack of systematic studies of patients with a more commonly seen partial proportion of decapitated spermatozoa.

In this study, we recruited 34 patients with acephalic spermatozoa syndrome and identified pathogenic variants in *ACTRT1* from two independent patients with partial acephalic spermatozoa syndrome. ACTRT1 is an actin-related protein that is mainly expressed in the calyx and PCM of bull spermatozoa (Heid et al., 2002). Similarly, we observed positive staining of ACTRT1 in the HTCA of human sperm, which suggests that ACTRT1 may affect the head-tail connection of spermatozoa by interacting with centrioles. Insertion mutations (c.547_548insA, p.Met183Asnfs^∗^17) have been identified in transcribed sequences encoding enhancer RNAs of *ACTRT1* in two families with Bazex-Dupré-Christol syndrome (Bal et al., 2017). Here, we identified the pathogenic variants, c.95G>A:p.Arg32His and c.662A>G: p Tyr221Cys, in the exon region of *ACTRT1* from patients F018/II:3 and F034/II:1, respectively. Both pathogenic variants caused amino acid substitutions and were predicted to be highly pathogenic. In addition, these rare pathogenic variants were found to be highly conserved among different species. In a three-dimensional protein model of ACTRT1, these pathogenic variants also showed a potential effect on the structure of the protein, which may further impair the function of HTCA. Moreover, we generated an *Actrt1*-knockout mouse model using CRISPR/Cas9 and found that most of the sperm from *Actrt1*-null mice were headless. Label-free quantitative proteomics analysis of the testis from *Actrt1*-null mice showed that the levels of approximately 132 proteins were increased and the levels of 112 proteins were decreased by more than 1.5 times. Gene ontology analysis showed that the proteins with altered expression levels were enriched in reproduction, cellular component organization or biogenesis, cell binding, and regulation of transport. The expression levels of Cep131 increased and the expression levels of the potential acephalic spermatozoa syndrome-related genes, *Tsga10* and *Brdt*, were significantly decreased. Therefore, defective *ACTRT1* in the PCM may lead to acephalic spermatozoa syndrome through its interaction with the constituent proteins of the HTCA. Thus, we speculated that defects in *ACTRT1* are a novel pathogenic mechanism of acephalic spermatozoa syndrome.

Sperm centrioles have multiple functions and play important roles in spermiogenesis (Avidor-Reiss et al., 2019). During this process, the axoneme is formed by the distal centrioles and it then extends to the end piece. Mutations in *CEP135*, which encodes a centrosomal protein, can cause multiple morphological abnormalities of the sperm flagella (Sha et al., 2017). Centrin1 expression leads to fibrous sheath dysplasia and head-neck junction anomalies (Moretti et al., 2017). Centrioles are an essential component of the HTCA and they play an important role in maintaining sperm integrity. Centriole-associated protein deficiencies or abnormalities often lead to acephalic spermatozoa syndrome. The deletion of *Spatc1l* (spermatogenesis and centriole-associated 1 like) alters the sperm head-tail integrity and causes acephalic spermatozoa syndrome in mice (Kim et al., 2018). Recently, we identified acephalic spermatozoa syndrome patients harboring *SPATC1L* mutations. Moreover, mutations in centrosomal protein 112 (*CEP112*) (Li et al., 2021) can also lead to acephalic spermatozoa syndrome in humans (Sha et al., 2020). ACTRT1 is highly expressed in the PCM of human spermatozoa. Therefore, mutations in *ACTRT1* are likely to affect HTCA function, resulting in acephalic spermatozoa syndrome.

Human sperm centrioles/centrosomes play crucial roles during fertilization and early embryonic development (Sathananthan et al., 1996; Fishman et al., 2018; Avidor-Reiss et al., 2019). The sperm centrosome contains a typical barrel-shaped proximal centriole, surrounding PCM, and an atypical distal centriole. The sperm provides the zygote with two centrioles, a typical proximal centriole, an atypical centriole attached to the axoneme base (the remodeled DC), a remodeled structural PCM, striated columns, and the capitulum (Fishman et al., 2018). Based on these components, the zygote initiates mitosis and forms the embryo. ACTRT1, a structural component of PCM, may play a role in fertilization and early human embryonic development. In this study, given that the spermatozoa of the patients were partially headless, we optimized the spermatozoa of the patients before performing artificial insemination for patients F018/II:3 and F034/II:1. With the optimized spermatozoa, successful clinical pregnancies and childbirth were achieved, which showed that artificial insemination with optimized spermatozoa is an effective treatment for patients with acephalic spermatozoa. Our results indicated that, although pathogenic variants of *ACTRT1* caused headless sperm, they did not affect the fertilization process. Intracytoplasmic sperm injection has been reported to be an effective treatment for patients with acephalic spermatozoa syndrome (Fang et al., 2018). However, artificial insemination is simpler and cheaper. Taken together, these data suggested that artificial insemination is an alternative treatment option for patients with acephalic spermatozoa syndrome.

With the increase in attention directed at reproductive health, there is a greater focus on researching the pathogenesis of acephalic spermatozoa syndrome. With the rapid development of next-generation sequencing technology, low-cost and rapid sequencing methods have allowed the genome-wide or exome-wide study on genetic defects in patients with acephalic spermatozoa syndrome (Krausz and Riera-Escamilla, 2018). Using WES, several pathogenic genes associated with acephalic spermatozoa syndrome have been identified in humans and animal models (Li et al., 2019). A systematic analysis of the genetic spectrum of human acephalic spermatozoa syndrome will be beneficial for the clinical screening and diagnosis of this disorder. Meanwhile, with a preimplantation genetic diagnosis and/or screening, acephalic spermatozoa syndrome may be prevented in the offspring of patients in the near future.

In summary, we identified pathogenic variants of *ACTRT1* in two idiopathic patients with acephalic spermatozoa syndrome. Both variants were deleterious and may affect the function and distribution of ACTRT1. *Actrt1*-knockout mice reproduced the phenotype of parallel acephalic spermatozoa. Therefore, our results demonstrated that pathogenic variants in *ACTRT1* are a novel pathogenic mechanism of acephalic spermatozoa syndrome. We also showed that an artificial insemination is a suitable option for patients with acephalic spermatozoa. This study provides further insights for clinicians and researchers into the genetic etiology and therapeutic strategies of acephalic spermatozoa syndrome.

## Data Availability Statement

The datasets presented in this study can be found in online repositories. The names of the repository/repositories and accession number(s) can be found below: China National GeneBank DataBase (CNGBdb): CNP0001834.

## Ethics Statement

The studies involving human participants were reviewed and approved by the Ethics Committees of the Women and Children’s Hospital of Xiamen University, the First Affiliated Hospital of Xiamen University, and Beijing Obstetrics and Gynecology Hospital. The patients/participants provided their written informed consent to participate in this study. The animal study was reviewed and approved by Laboratory Animal Management and Ethics Committee of Xiamen University.

## Author Contributions

YS, LL, YL, BZ, and YW recruited participants and analyzed the data. WL performed the experiments and wrote the manuscript. VI analyzed the data and revised the manuscript. MS performed the protein structural analysis. JC collected the clinical data. XW designed and supervised the entire project. All authors reviewed the manuscript.

## Conflict of Interest

The authors declare that the research was conducted in the absence of any commercial or financial relationships that could be construed as a potential conflict of interest.

## Publisher’s Note

All claims expressed in this article are solely those of the authors and do not necessarily represent those of their affiliated organizations, or those of the publisher, the editors and the reviewers. Any product that may be evaluated in this article, or claim that may be made by its manufacturer, is not guaranteed or endorsed by the publisher.

## Footnotes

1
http://broadinstitute.github.io/picard/

2
https://gatk.broadinstitute.org/hc/en-us

3
https://annovar.openbioinformatics.org

4
http://blocks.fhcrc.org/sift/SIFT.html

5
http://genetics.bwh.harvard.edu/pph2/

6
http://www.mutationtaster.org/

7
https://gnomad.broadinstitute.org/

8
https://swissmodel.expasy.org

9
https://spdbv.vital-it.ch/

## Supplementary Material

Supplementary Figure 1 Immunofluorescence co-staining of ACTRT1 and PCM1 on the sperm from a control subject. **(A)** The blue signal indicates the head of the sperm. **(B)** The green signal represents the expression of PCM1. **(C)** The red signal shows the expression of ACTRT1. **(D)** The merged image of PCM1 (green) and ACTRT1 (red). **(E)** The photograph of the sperm in the bright field. **(F)** The merged image of the sperm.

Supplementary Figure 2 Generation of *Actrt1* knockout mice. **(A)** Schematic diagram of generates the *Actrt* knockout mice by CRISPR-Cas9 system. **(B)** Polymerase Chain Reaction (PCR) identifies the genotype of *Actrt*-knockout mice. **(C)** Western blot analysis of the knockout efficiency of Actrt1-null mice.

Supplementary Figure 3 Spermatogenesis of *Actrt1* knockout mice. **(A)** HE staining of the testes from the wildtype and *Actrt1*-knockout mice. **(B)** HE staining of the epididymides from the wildtype and *Actrt1*-knockout mice.

Supplementary Figure 4 Percentage of headless sperm in the epididymides of the *Actrt1* knockout mice.

## References

[B1] Adzhubei I. Jordan D. M. Sunyaev S. R. (2013). Predicting functional effect of human missense mutations using PolyPhen-2. *Curr. Protoc. Hum. Genet.* 7:Unit720. 10.1002/0471142905.hg0720s76 23315928 PMC4480630

[B2] Avidor-Reiss T. Mazur M. Fishman E. L. Sindhwani P. (2019). The role of sperm centrioles in human reproduction - the known and the unknown. *Front. Cell Dev. Biol.* 7:188. 10.3389/fcell.2019.00188 31632960 PMC6781795

[B3] Baccetti B. Burrini A. G. Collodel G. Magnano A. R. Piomboni P. Renieri T. (1989). Morphogenesis of the decapitated and decaudated sperm defect in two brothers. *Gamete Res.* 23 181–188. 10.1002/mrd.1120230205 2731903

[B4] Bal E. Park H. S. Belaid-Choucair Z. Kayserili H. Naville M. Madrange M. (2017). Mutations in ACTRT1 and its enhancer RNA elements lead to aberrant activation of Hedgehog signaling in inherited and sporadic basal cell carcinomas. *Nat. Med.* 23 1226–1233. 10.1038/nm.4368 28869610

[B5] Chemes H. E. Carizza C. Scarinci F. Brugo S. Neuspiller N. Schwarsztein L. (1987). Lack of a head in human spermatozoa from sterile patients: a syndrome associated with impaired fertilization. *Fertil. Steril.* 47 310–316. 10.1016/s0015-0282(16)50011-9 3545911

[B6] Chemes H. E. Rawe V. Y. (2010). The making of abnormal spermatozoa: cellular and molecular mechanisms underlying pathological spermiogenesis. *Cell Tissue Res.* 341 349–357. 10.1007/s00441-010-1007-3 20596874

[B7] Chen H. Zhu Y. Zhu Z. Zhi E. Lu K. Wang X. (2018). Detection of heterozygous mutation in hook microtubule-tethering protein 1 in three patients with decapitated and decaudated spermatozoa syndrome. *J. Med. Genet.* 55 150–157. 10.1136/jmedgenet-2016-104404 29330334

[B8] Elkhatib R. A. Paci M. Longepied G. Saias-Magnan J. Courbiere B. Guichaoua M. R. (2017). Homozygous deletion of SUN5 in three men with decapitated spermatozoa. *Hum. Mol. Genet.* 26 3167–3171. 10.1093/hmg/ddx200 28541472

[B9] Fang J. Zhang J. Zhu F. Yang X. Cui Y. Liu J. (2018). Patients with acephalic spermatozoa syndrome linked to SUN5 mutations have a favorable pregnancy outcome from ICSI. *Hum. Reprod.* 33 372–377. 10.1093/humrep/dex382 29329387

[B10] Fawcett D. W. Phillips D. M. (1969). The fine structure and development of the neck region of the mammalian spermatozoon. *Anat. Rec.* 165 153–164. 10.1002/ar.1091650204 5387815

[B11] Fishman E. L. Jo K. Nguyen Q. P. H. Kong D. Royfman R. Cekic A. R. (2018). A novel atypical sperm centriole is functional during human fertilization. *Nat. Commun.* 9:2210. 10.1038/s41467-018-04678-8 29880810 PMC5992222

[B12] Heid H. Figge U. Winter S. Kuhn C. Zimbelmann R. Franke W. (2002). Novel actin-related proteins Arp-T1 and Arp-T2 as components of the cytoskeletal calyx of the mammalian sperm head. *Exp. Cell Res.* 279 177–187. 10.1006/excr.2002.5603 12243744

[B13] Hermo L. Pelletier R. M. Cyr D. G. Smith C. E. (2010). Surfing the wave, cycle, life history, and genes/proteins expressed by testicular germ cells. *Part 2: changes in spermatid organelles associated with development of spermatozoa*. *Microsc. Res. Tech.* 73 279–319. 10.1002/jemt.20787 19941292

[B14] Jiao S. Y. Yang Y. H. Chen S. R. (2021). Molecular genetics of infertility: loss-of-function mutations in humans and corresponding knockout/mutated mice. *Hum. Reprod. Update* 27 154–189. 10.1093/humupd/dmaa034 33118031

[B15] Kim J. Kwon J. T. Jeong J. Kim J. Hong S. H. Kim J. (2018). SPATC1L maintains the integrity of the sperm head-tail junction. *EMBO Rep.* 19:e45991. 10.15252/embr.201845991 30026308 PMC6123647

[B16] Koch L. (2020). Exploring human genomic diversity with gnomAD. *Nat. Rev. Genet.* 21:448. 10.1038/s41576-020-0255-7 32488197

[B17] Krausz C. Riera-Escamilla A. (2018). Genetics of male infertility. *Nat. Rev. Urol.* 15 369–384. 10.1038/s41585-018-0003-3 29622783

[B18] Li H. Durbin R. (2009). Fast and accurate short read alignment with Burrows-Wheeler transform. *Bioinformatics* 25 1754–1760. 10.1093/bioinformatics/btp324 19451168 PMC2705234

[B19] Li L. Sha Y. W. Xu X. Mei L. B. Qiu P. P. Ji Z. Y. (2018). DNAH6 is a novel candidate gene associated with sperm head anomaly. *Andrologia* 2018:12953. 10.1111/and.12953 29356036

[B20] Li L. Sha Y. Wang X. Li P. Wang J. Kee K. (2017). Whole-exome sequencing identified a homozygous BRDT mutation in a patient with acephalic spermatozoa. *Oncotarget* 8 19914–19922. 10.18632/oncotarget.15251 28199965 PMC5386733

[B21] Li L. Wang J. S. Yin C. H. Yue W. T. (2019). [Advances in the molecular genetic studies of acephalic spermatozoa syndrome]. *Zhonghua Nan Ke Xue* 25 838–842. 32233213

[B22] Li Y. Z. Li N. Liu W. S. Sha Y. W. Wu R. F. Tang Y. L. (2021). Biallelic mutations in spermatogenesis and centriole-associated 1 like (SPATC1L) cause acephalic spermatozoa syndrome and male infertility. *Asian J. Androl.* 10.4103/aja.aja_56_21 PMC8788604 34213489

[B23] Liu G. Wang N. Zhang H. Yin S. Dai H. Lin G. (2020). Novel mutations in PMFBP1. *TSGA10 and SUN5: Expanding the spectrum of mutations that may cause acephalic spermatozoa*. *Clin. Genet.* 97 938–939. 10.1111/cge.13747 32285443

[B24] Liu W. Sha Y. Li Y. Mei L. Lin S. Huang X. (2019). Loss-of-function mutations in SPEF2 cause multiple morphological abnormalities of the sperm flagella (MMAF). *J. Med. Genet.* 56 678–684. 10.1136/jmedgenet-2018-105952 31151990

[B25] Lu M. Kong S. Xiang M. Wang Y. Zhang J. Duan Z. (2021). A novel homozygous missense mutation of PMFBP1 causes acephalic spermatozoa syndrome. *J. Assist. Reprod. Genet.* 38 949–955. 10.1007/s10815-021-02075-7 33484382 PMC8079480

[B26] Mazaheri Moghaddam M. Mazaheri Moghaddam M. Hamzeiy H. Baghbanzadeh A. Pashazadeh F. Sakhinia E. (2021). Genetic basis of acephalic spermatozoa syndrome, and intracytoplasmic sperm injection outcomes in infertile men: a systematic scoping review. *J. Assist. Reprod. Genet.* 38 573–586. 10.1007/s10815-020-02008-w 33452591 PMC7910383

[B27] McKenna A. Hanna M. Banks E. Sivachenko A. Cibulskis K. Kernytsky A. (2010). The Genome Analysis Toolkit: a MapReduce framework for analyzing next-generation DNA sequencing data. *Genome Res.* 20 1297–1303. 10.1101/gr.107524.110 20644199 PMC2928508

[B28] Moretti E. Pascarelli N. A. Belmonte G. Renieri T. Collodel G. (2017). Sperm with fibrous sheath dysplasia and anomalies in head-neck junction: focus on centriole and centrin 1. *Andrologia* 49:12701. 10.1111/and.12701 27596234

[B29] Ng P. C. Henikoff S. (2003). SIFT: Predicting amino acid changes that affect protein function. *Nucleic Acids Res.* 31 3812–3814. 10.1093/nar/gkg509 12824425 PMC168916

[B30] Perotti M. E. Giarola A. Gioria M. (1981). Ultrastructural study of the decapitated sperm defect in an infertile man. *J. Reprod. Fertil.* 63 543–549. 10.1530/jrf.0.0630543 7299757

[B31] Reina J. Gottardo M. Riparbelli M. G. Llamazares S. Callaini G. Gonzalez C. (2018). Centrobin is essential for C-tubule assembly and flagellum development in Drosophila melanogaster spermatogenesis. *J. Cell Biol.* 217 2365–2372. 10.1083/jcb.201801032 29712734 PMC6028543

[B32] Sathananthan A. H. Ratnam S. S. Ng S. C. Tarin J. J. Gianaroli L. Trounson A. (1996). The sperm centriole: its inheritance, replication and perpetuation in early human embryos. *Hum. Reprod.* 11 345–356. 10.1093/humrep/11.2.345 8671223

[B33] Schwarz J. M. Rodelsperger C. Schuelke M. Seelow D. (2010). MutationTaster evaluates disease-causing potential of sequence alterations. *Nat. Methods* 7 575–576. 10.1038/nmeth0810-575 20676075

[B34] Sha Y. W. Sha Y. K. Ji Z. Y. Mei L. B. Ding L. Zhang Q. (2018a). TSGA10 is a novel candidate gene associated with acephalic spermatozoa. *Clin. Genet.* 93 776–783. 10.1111/cge.13140 28905369

[B35] Sha Y. W. Wang X. Xu X. Ding L. Liu W. S. Li P. (2019). Biallelic mutations in PMFBP1 cause acephalic spermatozoa. *Clin. Genet.* 95 277–286. 10.1111/cge.13461 30298696

[B36] Sha Y. W. Xu X. Ji Z. Y. Lin S. B. Wang X. Qiu P. P. (2018b). Genetic contribution of SUN5 mutations to acephalic spermatozoa in Fujian China. *Gene* 647 221–225. 10.1016/j.gene.2018.01.035 29331481

[B37] Sha Y. W. Xu X. Mei L. B. Li P. Su Z. Y. He X. Q. (2017). A homozygous CEP135 mutation is associated with multiple morphological abnormalities of the sperm flagella (MMAF). *Gene* 633 48–53. 10.1016/j.gene.2017.08.033 28866084

[B38] Sha Y. Wang X. Yuan J. Zhu X. Su Z. Zhang X. (2020). Loss-of-function mutations in centrosomal protein 112 is associated with human acephalic spermatozoa phenotype. *Clin. Genet.* 97 321–328. 10.1111/cge.13662 31654588

[B39] Shang Y. Zhu F. Wang L. Ouyang Y. C. Dong M. Z. Liu C. (2017). Essential role for SUN5 in anchoring sperm head to the tail. *Elife* 6:28199. 10.7554/eLife.28199 28945193 PMC5634783

[B40] Vaser R. Adusumalli S. Leng S. N. Sikic M. Ng P. C. (2016). SIFT missense predictions for genomes. *Nat. Protoc.* 11 1–9. 10.1038/nprot.2015.123 26633127

[B41] Wang K. Li M. Hakonarson H. (2010). ANNOVAR: functional annotation of genetic variants from high-throughput sequencing data. *Nucleic Acids Res.* 38:e164. 10.1093/nar/gkq603 20601685 PMC2938201

[B42] Wu B. Gao H. Liu C. Li W. (2020). The coupling apparatus of the sperm head and tail. *Biol. Reprod.* 102 988–998. 10.1093/biolre/ioaa016 31995163

[B43] Ye Y. Wei X. Sha Y. Li N. Yan X. Cheng L. (2020). Loss-of-function mutation in TSGA10 causes acephalic spermatozoa phenotype in human. *Mol. Genet. Genomic Med.* 8:e1284. 10.1002/mgg3.1284 32410354 PMC7336754

[B44] Zhang D. Huang W. J. Chen G. Y. Dong L. H. Tang Y. Zhang H. (2021). Pathogenesis of acephalic spermatozoa syndrome caused by SUN5 variant. *Mol. Hum. Reprod.* 27:gaab028. 10.1093/molehr/gaab028 33848337

[B45] Zhu F. Liu C. Wang F. Yang X. Zhang J. Wu H. (2018). Mutations in PMFBP1 Cause acephalic spermatozoa syndrome. *Am. J. Hum. Genet.* 103 188–199. 10.1016/j.ajhg.2018.06.010 30032984 PMC6080767

[B46] Zhu F. Wang F. Yang X. Zhang J. Wu H. Zhang Z. (2016). Biallelic SUN5 Mutations Cause Autosomal-Recessive Acephalic Spermatozoa Syndrome. *Am. J. Hum. Genet.* 99:1405. 10.1016/j.ajhg.2016.11.002 27912045 PMC5142114

