# Supplementary figures and images for: Pathogenic Variants in ACTRT1 Cause Acephalic Spermatozoa Syndrome
Source: Front Cell Dev Biol. 2021 Aug 6;9:676246. doi: 10.3389/fcell.2021.676246 (PMC8377740; doi:10.3389/fcell.2021.676246)

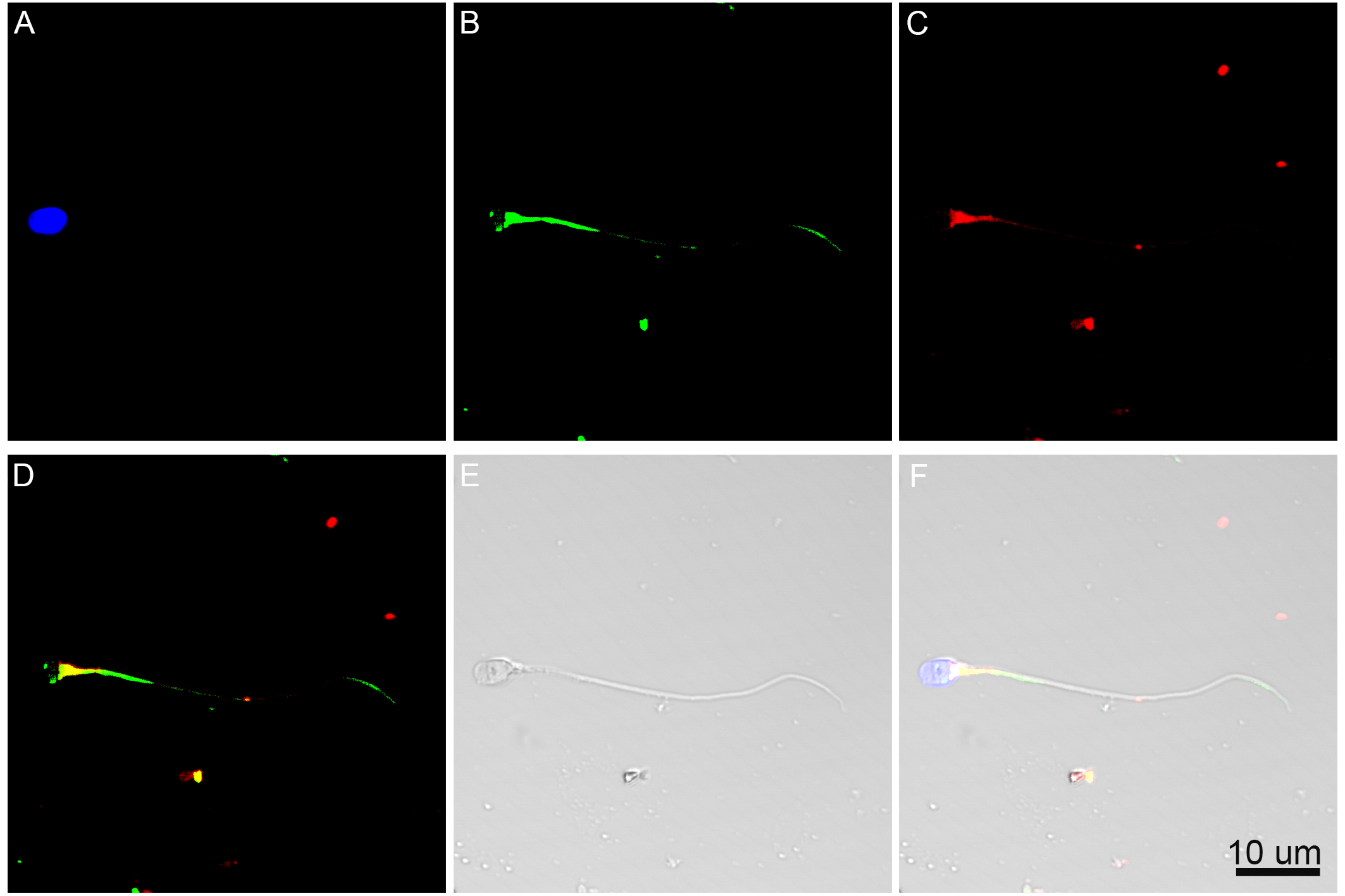

Supplement: Supplementary Figure 1 — Immunofluorescence co-staining of ACTRT1 and PCM1 on the sperm from a control subject. (A) The blue signal indicates the head of the sperm. (B) The green signal represents the expression of PCM1. (C) The red signal shows the expression of ACTRT1. (D) The merged image of PCM1 (green) and ACTRT1 (red). (E) The photograph of the sperm in the bright field. (F) The merged image of the sperm. [file Image_1.TIF]

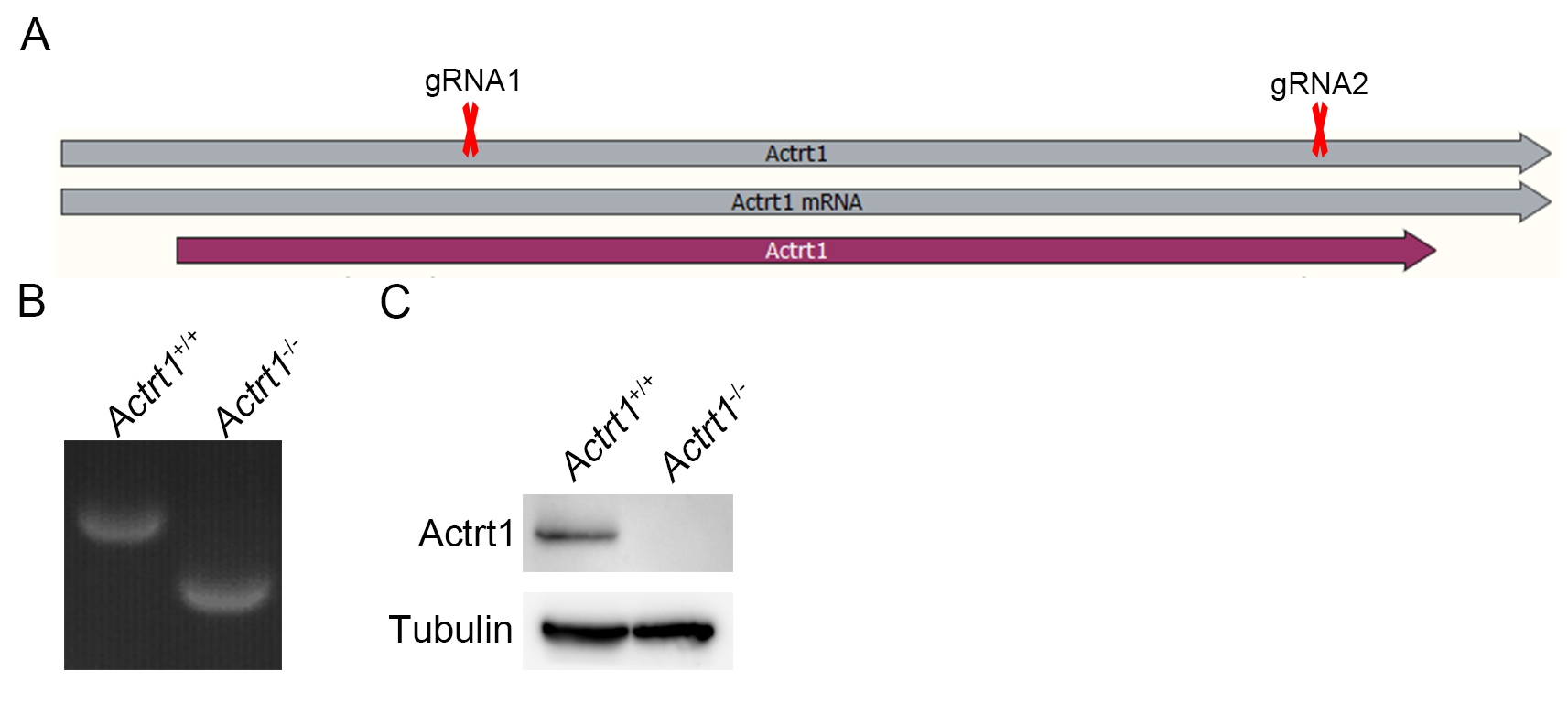

Supplement: Supplementary Figure 2 — Generation of Actrt1 knockout mice. (A) Schematic diagram of generates the Actrt knockout mice by CRISPR-Cas9 system. (B) Polymerase Chain Reaction (PCR) identifies the genotype of Actrt-knockout mice. (C) Western blot analysis of the knockout efficiency of Actrt1-null mice. [file Image_2.TIF]

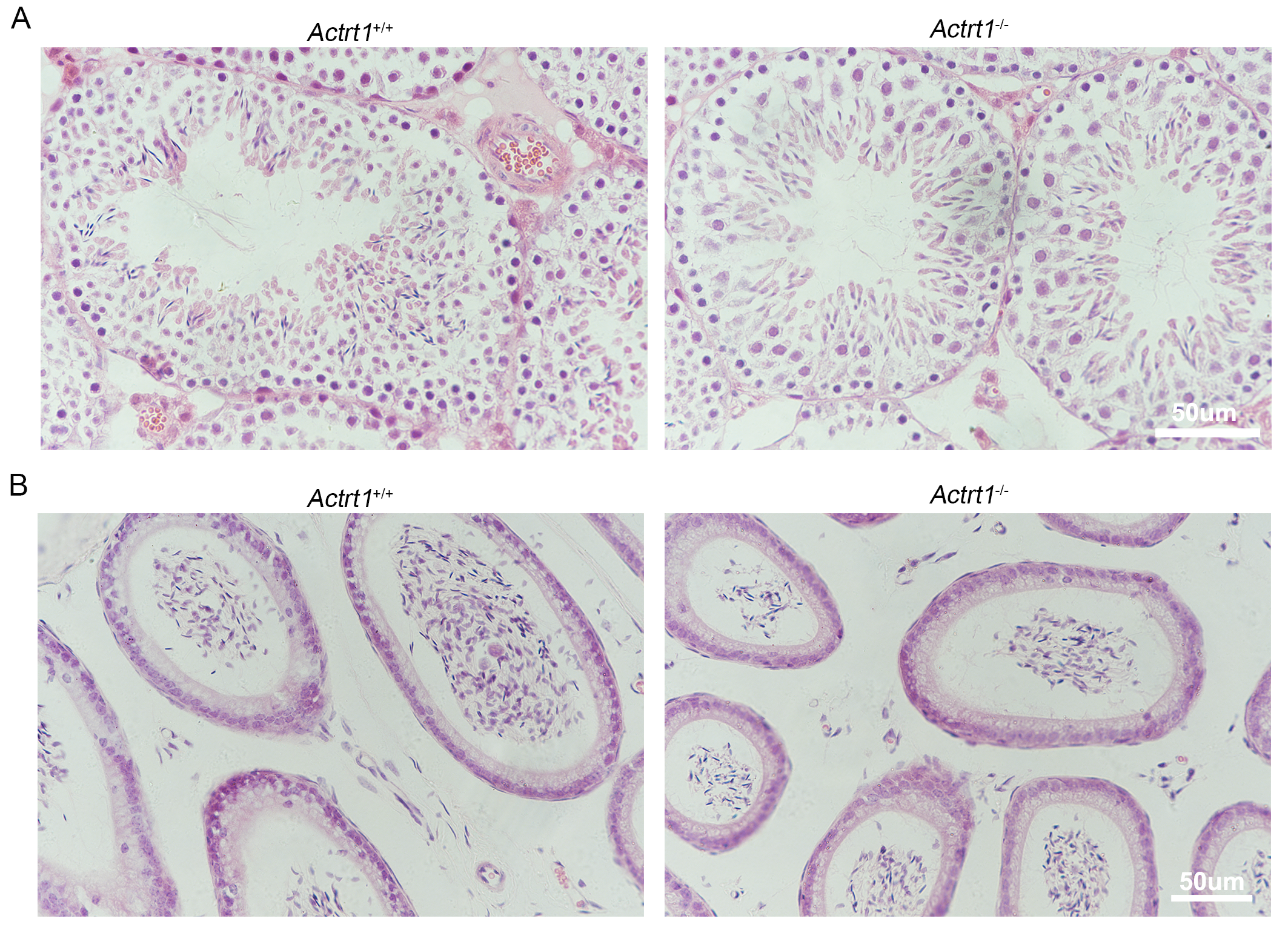

Supplement: Supplementary Figure 3 — Spermatogenesis of Actrt1 knockout mice. (A) HE staining of the testes from the wildtype and Actrt1-knockout mice. (B) HE staining of the epididymides from the wildtype and Actrt1-knockout mice. [file Image_3.TIF]

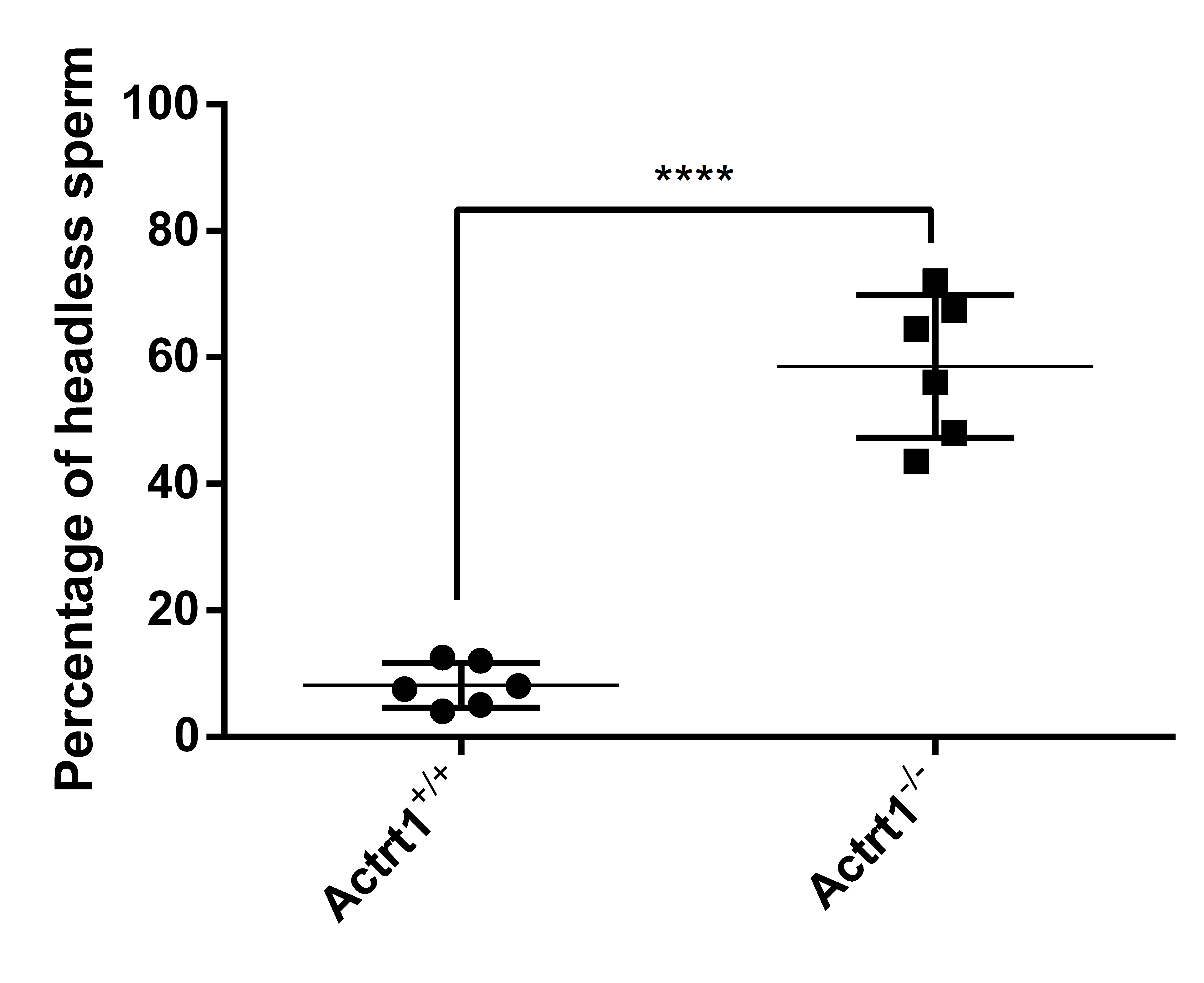

Supplement: Supplementary Figure 4 — Percentage of headless sperm in the epididymides of the Actrt1 knockout mice. [file Image_4.TIF]
