# Supplementary material for: Pathogenic Variants in ACTRT1 Cause Acephalic Spermatozoa Syndrome
Source: Front Cell Dev Biol. 2021 Aug 6;9:676246. doi: 10.3389/fcell.2021.676246 (PMC8377740; doi:10.3389/fcell.2021.676246)
Supplement: Supplementary file 5 [file Data_Sheet_1.docx]

**Supplementary Table 1. PCR primers for Sanger sequencing**

| Target | Forward | Reverse |
| --- | --- | --- |
| NM_138289.4:c.95G>A | CTGGTGGACTGAAGGTAGCC | GTTTTACTCCAAGCTCCCGC |
| NM_138289.4: c.662A>G | CTATGCCTCTGCCTGTGTCA | GAAGCCAGCTGTTCCACTTC |

**Supplementary Table 2: Clinical data of the patients with partial acephalic spermatozoa**.

| **Patient** | F018/II:3 | F034/II:1 | **Reference** |
| --- | --- | --- | --- |
| Age (year) | 30 | 33 | - |
| Height (cm) | 172 | 177 | - |
| Body weight (kg) | 65 | 76 | - |
| Infertility （year） | 2 | 3 | - |
| Testicular volume (Left/Right) | 12/12 | 12/12 | 10-15ml |
| FSH（mIU/ml） | 11.92 | 9.72 | 1.27～18.96 |
| LH（mIU/ml） | 5.24 | 6.44 | 1.24～8.62 |
| Testosterone (ng/ml) | 6.75 | 5.39 | 4.14-7.26 |
| PRL (ng/ml) | 11.26 | 8.65 | 2.64～13.13 |
| E2 (pg/ml) | 43 | 31 | 20～75 |

**Supplementary Table 3: Fertility measurement of *Actrt1*-knockout mice**

| **Male (n=6)** | **Litter times** | **Pups** | **Litter Size (mean ± SD)** |
| --- | --- | --- | --- |
| *Actrt1*^+/+^ | 29 | 231 | 7.96±1.88 |
| *Actrt1*^-/-^ | 24 | 91 | 3.14±1.73 |

**Supplementary Table 4: Proteins with a change more than two times.**

| Gene | Protein Name | *Actrt1*^+/+^ /*Actrt1*^-/-^ | P value | FDR value |
| --- | --- | --- | --- | --- |
| Rmnd1 | Required for meiotic nuclear division protein 1 homolog | 11.9327 | 0.0015 | 0.0057 |
| Pon2 | Serum paraoxonase/arylesterase 2 | 8.9877 | 0.0103 | 0.0108 |
| Vps53 | Vacuolar protein sorting-associated protein 53 homolog | 6.4083 | 0.0345 | 0.0333 |
| Daam2 | Disheveled-associated activator of morphogenesis 2 | 4.8237 | 0.0003 | 0.0003 |
| Mrps34 | 28S ribosomal protein S34, mitochondrial | 4.4931 | 0.0003 | 0.0518 |
| Znf512 | Zinc finger protein 512 | 4.2588 | 0.0005 | 0.0447 |
| Retreg3 | Reticulophagy regulator 3 | 3.8767 | 0.0010 | 0.0074 |
| Colq | Acetylcholinesterase collagenic tail peptide | 3.6920 | 0.0008 | 0.0001 |
| Spata3 | Spermatogenesis-associated protein 3 | 3.1221 | 0.0008 | 0.0071 |
| Slc48a1 | Heme transporter HRG1 | 2.9491 | 0.0013 | 0.0193 |
| Sri | Sorcin | 2.8613 | 0.0017 | 0.0579 |
| Klhl22 | Kelch-like protein 22 | 2.8294 | 0.0440 | 0.0001 |
| Mydgf | Myeloid-derived growth factor | 2.6469 | 0.0002 | 0.0128 |
| Eln | Elastin | 2.5148 | 0.0003 | 0.0621 |
| Mtcl1 | Microtubule cross-linking factor 1 | 2.5138 | 0.0005 | 0.0002 |
| Kiaa1324 | UPF0577 protein KIAA1324 | 2.3853 | 0.0076 | 0.0071 |
| Isca2 | Iron-sulfur cluster assembly 2 homolog, mitochondrial | 2.3834 | 0.0011 | 0.0053 |
| Pycard | Apoptosis-associated speck-like protein containing a CARD | 2.3737 | 0.0226 | 0.0014 |
| Col4a1 | Collagen alpha-1(IV) chain | 2.3662 | 0.0063 | 0.1059 |
| Entpd2 | Ectonucleoside triphosphate diphosphohydrolase 2 | 2.3428 | 0.0007 | 0.1795 |
| Abca1 | Phospholipid-transporting ATPase ABCA1 | 2.3188 | 0.0033 | 0.0007 |
| Abcc12 | Multidrug resistance-associated protein 9 | 2.2813 | 0.0467 | 0.0004 |
| Atp6v1e1 | V-type proton ATPase subunit E 1 | 2.2508 | 0.0007 | 0.0164 |
| Col4a3 | Collagen alpha-3(IV) chain | 2.1943 | 0.0002 | 0.0638 |
| Ltbp1 | Latent-transforming growth factor beta-binding protein 1 | 2.1531 | 0.0022 | 0.0590 |
| Rala | Ras-related protein Ral-A | 2.1495 | 0.0207 | 0.0083 |
| Col4a4 | Collagen alpha-4(IV) chain | 2.1333 | 0.0003 | 0.0436 |
| Polq | DNA polymerase theta | 2.1194 | 0.0092 | 0.0308 |
| Col4a2 | Collagen alpha-2(IV) chain | 2.1171 | 0.0396 | 0.2289 |
| Sox4 | Transcription factor SOX-4 | 2.0632 | 0.0007 | 0.0019 |
| Ep300 | Histone acetyltransferase p300 | 2.0330 | 0.0074 | 0.0002 |
| B4galnt1 | Beta-1,4 N-acetylgalactosaminyltransferase 1 | 2.0187 | 0.0030 | 0.0095 |
| Micu2 | Calcium uptake protein 2, mitochondrial | 2.0138 | 0.0007 | 0.0016 |
| Rrp1b | Ribosomal RNA processing protein 1 homolog B | 2.0028 | 0.0003 | 0.0524 |
| Usp26 | Ubiquitin carboxyl-terminal hydrolase 26 | 0.1263 | 0.0004 | 0.0138 |
| Spag7 | Sperm-associated antigen 7 | 0.1502 | 0.0021 | 0.0010 |
| Cfap43 | Cilia- and flagella-associated protein 43 | 0.1570 | 0.0037 | 0.1934 |
| Kank2 | KN motif and ankyrin repeat domain-containing protein 2 | 0.1695 | 0.0003 | 0.0649 |
| Cyp2d11 | Cytochrome P450 2D11 | 0.1861 | 0.0008 | 0.0058 |
| Spg7 | Paraplegin | 0.1889 | 0.0158 | 0.0277 |
| Tti1 | TELO2-interacting protein 1 homolog | 0.2353 | 0.0068 | 0.0393 |
| Efr3a | Protein EFR3 homolog A | 0.2517 | 0.0151 | 0.0087 |
| Ccdc160 | Coiled-coil domain-containing protein 160 | 0.2560 | 0.0297 | 0.0024 |
| Chmp7 | Charged multivesicular body protein 7 | 0.2629 | 0.0003 | 0.0013 |
| Mfap4 | Microfibril-associated glycoprotein 4 | 0.2631 | 0.0002 | 0.0135 |
| Tmem14c | Transmembrane protein 14C | 0.2864 | 0.0004 | 0.0060 |
| Unc79 | Protein unc-79 homolog | 0.3083 | 0.0011 | 0.0017 |
| Nufip2 | Nuclear fragile X mental retardation-interacting protein 2 | 0.3162 | 0.0009 | 0.0012 |
| Krt76 | Keratin, type II cytoskeletal 2 oral | 0.3187 | 0.0003 | 0.0030 |
| Qsox2 | Sulfhydryl oxidase 2 | 0.3231 | 0.0003 | 0.0004 |
| Tmx2 | Thioredoxin-related transmembrane protein 2 | 0.3264 | 0.0002 | 0.0045 |
| Rabep2 | Rab GTPase-binding effector protein 2 | 0.3391 | 0.0053 | 0.0016 |
| Tbc1d1 | TBC1 domain family member 1 | 0.3404 | 0.0004 | 0.0066 |
| Mrgbp | MRG/MORF4L-binding protein | 0.3433 | 0.0009 | 0.0002 |
| Rnf2 | E3 ubiquitin-protein ligase RING2 | 0.3435 | 0.0011 | 0.0252 |
| Pex11a | Peroxisomal membrane protein 11A | 0.3642 | 0.0094 | 0.0539 |
| Pcid2 | PCI domain-containing protein 2 | 0.3643 | 0.0029 | 0.0103 |
| Sh3glb2 | Endophilin-B2 | 0.3693 | 0.0038 | 0.0007 |
| Dtd2 | D-aminoacyl-tRNA deacylase 2 | 0.3707 | 0.0462 | 0.0920 |
| Nsmce3 | Non-structural maintenance of chromosomes element 3 homolog | 0.3719 | 0.0074 | 0.1983 |
| Col3a1 | Collagen alpha-1(III) chain | 0.3746 | 0.0069 | 0.0711 |
| Ddx3y | ATP-dependent RNA helicase DDX3Y | 0.3774 | 0.0015 | 0.0086 |
| Tmed1 | Transmembrane emp24 domain-containing protein 1 | 0.3862 | 0.0476 | 0.0983 |
| Tspan6 | Tetraspanin-6 | 0.3916 | 0.0419 | 0.0126 |
| Ddb2 | DNA damage-binding protein 2 | 0.3932 | 0.0011 | 0.0738 |
| Polr2l | DNA-directed RNA polymerases I, II, and III subunit RPABC5 | 0.4010 | 0.0179 | 0.0001 |
| Sec24a | Protein transport protein Sec24A | 0.4031 | 0.0002 | 0.0002 |
| Ccny | Cyclin-Y | 0.4057 | 0.0051 | 0.0027 |
| Hat1 | Histone acetyltransferase type B catalytic subunit | 0.4066 | 0.0074 | 0.0949 |
| Tasor2 | Protein TASOR 2 | 0.4248 | 0.0106 | 0.0045 |
| Eml4 | Echinoderm microtubule-associated protein-like 4 | 0.4252 | 0.0062 | 0.0948 |
| Rpl37a | 60S ribosomal protein L37a | 0.4288 | 0.0247 | 0.0078 |
| Dhx32 | Putative pre-mRNA-splicing factor ATP-dependent RNA helicase DHX32 | 0.4296 | 0.0135 | 0.0385 |
| Tsc2 | Tuberin | 0.4314 | 0.0175 | 0.0268 |
| Vps45 | Vacuolar protein sorting-associated protein 45 | 0.4324 | 0.0014 | 0.0555 |
| Uqcr11 | Cytochrome b-c1 complex subunit 10 | 0.4354 | 0.0054 | 0.5684 |
| Ddx27 | Probable ATP-dependent RNA helicase DDX27 | 0.4372 | 0.0331 | 0.0275 |
| Nup85 | Nuclear pore complex protein Nup85 | 0.4372 | 0.0004 | 0.2113 |
| Tm9sf2 | Transmembrane 9 superfamily member 2 | 0.4380 | 0.0054 | 0.1647 |
| Fam53c | Protein FAM53C | 0.4419 | 0.0003 | 0.1589 |
| Gfm2 | Ribosome-releasing factor 2, mitochondrial | 0.4478 | 0.0178 | 0.0035 |
| Atp6v0a1 | V-type proton ATPase 116 kDa subunit a isoform 1 | 0.4536 | 0.0105 | 0.1053 |
| Wasl | Neural Wiskott-Aldrich syndrome protein | 0.4599 | 0.0003 | 0.0288 |
| Ttn | Titin | 0.4601 | 0.0002 | 0.1825 |
| Rnf180 | E3 ubiquitin-protein ligase RNF180 | 0.4617 | 0.0092 | 0.0531 |
| Smarcd1 | SWI/SNF-related matrix-associated actin-dependent regulator of chromatin subfamily D member 1 | 0.4625 | 0.0007 | 0.1288 |
| Rnf151 | RING finger protein 151 | 0.4668 | 0.0002 | 0.0045 |
| Npepl1 | Probable aminopeptidase NPEPL1 | 0.4766 | 0.0003 | 0.0831 |
| Mpp6 | MAGUK p55 subfamily member 6 | 0.4776 | 0.0051 | 0.1131 |
| Ddx3x | ATP-dependent RNA helicase DDX3X | 0.4861 | 0.0002 | 0.0735 |
| Homer1 | Homer protein homolog 1 | 0.4878 | 0.0002 | 0.0073 |
| Meiob | Meiosis-specific with OB domain-containing protein | 0.4886 | 0.0003 | 0.0002 |
| Tmco1 | Calcium load-activated calcium channel | 0.4956 | 0.0333 | 0.0940 |
| Otud4 | OTU domain-containing protein 4 | 0.4963 | 0.0010 | 0.0430 |
| Tsr1 | Pre-rRNA-processing protein TSR1 homolog | 0.4978 | 0.0002 | 0.0494 |
| Adgb | Androglobin | 0.4983 | 0.0142 | 0.1983 |
| Armc6 | Armadillo repeat-containing protein 6 | 0.4990 | 0.0071 | 0.0227 |

**Supplementary Table 5: Changes in proteins associated with acephalic spermatozoa syndrome.**

| Gene | Protein Name | *Actrt1*^+/+^/*Actrt1*^-/-^ | P value | FDR value |
| --- | --- | --- | --- | --- |
| Hook1 | Protein Hook homolog 1 | 1.2513 | 0.0081 | 0.1206 |
| Odf1 | Outer dense fiber protein 1 | 1.2460 | 0.0016 | 0.0383 |
| Spatc1l | Speriolin-like protein | 1.2160 | 0.0078 | 0.0681 |
| Spata6 | Spermatogenesis-associated protein 6 | 1.1248 | 0.0172 | 0.0351 |
| Ift88 | Intraflagellar transport protein 88 homolog | 1.0820 | 0.0097 | 0.0030 |
| Brdt | Bromodomain testis-specific protein | 0.6795 | 0.0072 | 0.0421 |
| Tsga10 | Testis-specific gene 10 protein | 0.6186 | 0.0011 | 0.0022 |
